# Complete Genome Sequence of Staphylococcus arlettae AHKW2e, Isolated from a Dog’s Paws in Hong Kong

**DOI:** 10.1128/mra.00350-22

**Published:** 2022-06-27

**Authors:** A. H. K. Wong, G. K. K. Lai, S. D. J. Griffin, F. C. C. Leung

**Affiliations:** a Shuyuan Molecular Biology Laboratory, The Independent Schools Foundation Academy, Hong Kong SAR, China; University of Rochester School of Medicine and Dentistry

## Abstract

Staphylococcus arlettae is commonly found on the skin of animals. Here, we describe the complete genome sequence of *S. arlettae* AHKW2e (2,649,260 bp; GC content, 33.6%), isolated from a dog’s paws in Hong Kong, established through hybrid assembly and representing the second complete genome sequence of this species.

## ANNOUNCEMENT

Staphylococcus arlettae is a Gram-positive, coagulase-negative, coccoid bacterium that was first isolated from the skin and nares of poultry and goats (1) and subsequently, most often from the udders of cows (2, 3). It is only a rare cause of infection in humans (4), and despite concerns that it may constitute an emerging opportunistic pathogen (5), strains of *S. arlettae* and other coagulase-negative staphylococci have been found to exhibit growth-inhibiting and quorum-quenching activity against S. aureus (6, 7). Among non-*aureus* staphylococci, antimicrobial-resistance (AMR) genes may be both plasmid borne and integrated into the chromosome via mobile elements (8); they have been frequently detected in strains of *S. arlettae* obtained from farm animals (9–11).

Strain AHKW2e was recovered from swab samples of a dog’s paws (a 4-year-old Goldendoodle) in Hong Kong, within a broader survey of bacterial diversity. The samples were rinsed with 1.5 mL of 0.9% (wt/vol) saline, and swabs of these extracts were applied to Luria agar and incubated overnight at 37°C. Colonies with different bacterial morphology and antibiotic resistance profiles (assessed by growth on Luria agar containing 0.01% (wt/vol) ampicillin, tetracycline, or kanamycin) were selected for genomic sequencing. AHKW2e (which formed circular, entire, raised, pale-yellow, ampicillin-resistant colonies) was passaged 10 times on Luria agar and finally incubated overnight in Luria broth (all at 37°C) (12) before DNA extraction using Invitrogen’s PureLink genomic DNA minikit, following the manufacturer’s instructions. Paired-end short-read sequencing libraries were prepared using the Nextera XT DNA library preparation kit and sequenced via the Illumina MiSeq platform with v3 chemistry (2 × 300 bp). Adapter sequences were removed using Trimmomatic v0.32 (13) and the reads quality filtered and trimmed. The resulting Illumina data set contained 1,016,145 read pairs with an average length of 296 bp (~301 Mbp). Long-read libraries, prepared from the same extracted DNA using the rapid barcoding kit (SQK-RBK004), were sequenced using a SpotON R9 flow cell and MinION sequencer, with data acquisition using MinKNOW v3.1.8 software and base calling using Guppy v2.1.3 (all from Oxford Nanopore). The final long-read data set, trimmed using Porechop v0.2.4 (14, 15), totaled 495,082 reads (2.31 Gbp) with a median length of 3,202 bp (*N*_50_, 7,626 bp). Default parameters were used for all software unless otherwise specified.

The complete genome sequence, assembled by combining the Illumina and MinION datasets using Unicycler v0.4.3 (16), comprises a circular chromosome of 2,606,218 bp (GC content, 33.63%) and a circular plasmid of 43,042 bp (GC content, 31.20%), with a genome coverage of 189×. The sequence was submitted to NCBI PGAP v5.0 for annotation (17).

The chromosome has an average nucleotide identity (using OrthoANIu at https://www.ezbiocloud.net/tools/ani) of 99.06% with Staphylococcus arlettae P2 (GenBank accession number AP019698) (18, 19). It contains the species-specific beta-lactamase *bla*_ARL-2_ (20, 21) and the metal resistance genes *arsDARBC*, *cadCAD*, *merA*, and *copB2-mco* as a large cluster. Plasmid pAHKW2e contains the *copAZ-csoR* operon, together with other environmental adaptation genes, including *qacC* and the formaldehyde-inducible *hxlAB* operon (22). Three QacE-like transporters are chromosomally encoded (23).

### Data availability.

The complete genome sequences and raw sequence data for Staphylococcus arlettae AHKW2e are available through NCBI under BioProject accession number PRJNA758761, GenBank accession numbers CP082336.1 (chromosome) and CP082337.1 (plasmid), and SRA accession numbers SRX12277472 (MiSeq) and SRX12151937 (MinION).
